# Medical Ethnobiology and Ethnopharmacology in Latin America

**DOI:** 10.1155/2012/379160

**Published:** 2012-03-19

**Authors:** Ulysses Paulino Albuquerque, Edwin L. Cooper, Maria Franco Trindade Medeiros, Rômulo Romeu Nóbrega Alves, Ana H. Ladio

**Affiliations:** ^1^Laboratory of Applied Ethnobotany, Department of Biology, Federal Rural University of Pernambuco, 52171-900 Recife, PE, Brazil; ^2^Laboratory of Comparative Neuroimmunology, Department of Neurobiology, University of California, Los Angeles, Los Angeles, CA 90095, USA; ^3^Ethnozoology, Conservation and Biodiversity Research Group, Department of Biology, State University of Paraíba, 58429-500 João Pessoa, PB, Brazil; ^4^Laboratorio Ecotono, Universidad Nacional del Comahue, Quintral, Río Negro 1250-8400 San Carlos de Bariloche, Argentina

Through their long exposure and experience with natural resources, many local communities in Latin America have developed health care practices. Thousands of years of observation and experimentation have helped in developing different empirical medical systems, as well as knowledge of plants, animals, and minerals. Such knowledge is the subject of medical ethnobiology and ethnopharmacology, disciplines that before being exclusive actually complemented each other. In the broadest sense, both medical ethnobiology and ethnopharmacology attempt to make sense and to understand traditional medical systems: the first from perceptions, healing strategies, natural resources used to fight diseases or maintain health; the second from traditional medicines, either plants, animals, or minerals.

We can find new and different types of approaches and theoretical and methodological developments such as ethnopharmacological evaluations of traditional drugs unknown so far; the inclusion of historical perspective in ethnopharmacological studies; the migration influence on traditional medical systems both in industrialized countries and remote locations, or a greater focus on urban contexts in ethnopharmacology. Moreover, the integrative aspect is noteworthy; it includes medical ethnobotany and zootherapy (the treatment of human diseases using drug-based therapies derived from animals).

Recent developments in methods and theory, like any evolving discipline, promote discussions of theoretical scenarios and help us understand the contexts of traditional medical systems as well as methods and techniques that enable access to these systems. Perhaps, a very common approach has been the development of quantitative techniques to access information about animals and plants used in these medical systems, in order to constitute a way to objectively select resources for phytochemical and pharmacological studies. Ethnopharmacological evaluations of traditional drugs is perhaps an approach that concentrates most investigations, focusing on an assessment of traditional preparations regarding their effectiveness. Both animals and plants provide extensive resources for new Complementary and Alternative Medicine (CAM) approaches which may prove important for future applications. Despite criticism about errors in experimentation and data interpretation this approach has proven useful. 

The historical perspective of ethnopharmacology has focused on the medicinal use of natural products that has preceded recorded human history probably by thousands of years. Surveys of those medicaments used in the past show that whereas compounders of medicines have invariably used vegetable, animal, and mineral substances, animals are less prevalent than herbs, and more prevalent than minerals. Historical texts showed that the treatment of illnesses using animal-based remedies is an extremely old practice. Animal-based remedies have constituted part of the inventory of medicinal substances used in various cultures since ancient times. Until now, studies concerning diversity of plants and animals used in medical systems of immigrants have occupied little space in the ethnopharmacological literature. Thus there has been influence of migration on traditional medical systems. Ethnobiological studies have shown that the natural product diversity used by people arises from a number of learning strategies, both simple and complex and that sophisticated social learning in particular plays a key role in transmitting variation in behavior between generations. 

Medical ethnobotany and zootherapy constitutes an important alternative among many other known therapies practiced worldwide. There is growing recognition that people in different parts of the world still use animal-and plant-based remedies as primary or complementary medicine. Probably the most famous of these are the Chinese, who use animals for a variety of ailments. Lesser known and studied, though just as varied and rich is Latin America's and Africa long tradition of animal remedies for all kinds of ailments. For example, in Traditional Chinese Medicine, more than 1500 animal species have been recorded to be of some medicinal use. In Brazil, at least 326 medicinal animals have been recorded, and at least 584 animal species, in Latin America. Using plants and animals for medicinal purposes is part of a body of traditional knowledge which is becoming increasingly more relevant to discussions on conservation biology, public health policies, and sustainable management of natural resources, biological prospection, and patents. Ethnopharmacology in urban contexts while expanding in most of the world continue to supplement limited public health facilities and more expensive commercially produced medications with popular remedies; this has led to an increasing demand for wildlife products for medicinal purposes in urban areas. A reflection is the widespread trade in medicinal plants and animals, mainly concentrated in local and traditional market in urban areas.

This Special Issue of 22 peer-reviewed papers include in this collection various dimensions of the constitutional process of healing practices through the use of plants and animals that local communities in Latin America developed over centuries of experimentation. The papers explore different aspects of the empirical use of natural resources including the cultural dimensions that influence the extraction of natural products, evaluation of medicinal efficacy of these products; intermedical character of traditional medical systems; application of fossils in folk medicine. All these researches highlight the importance of local perceptions and knowledge as potential information that can contribute to future applications and therefore as a new source of medicines from natural products.

